# Differential Expression of Immunohistochemical Markers in Ameloblastoma & Ameloblastic Carcinoma: A Systematic Review and Meta-analysis of observational studies

**DOI:** 10.12688/f1000research.149861.1

**Published:** 2024-05-31

**Authors:** Saleena Mishra, Swagatika Panda, Neeta Mohanty, Swati Mishra, Divya Gopinath, Saurav Panda, Sukumaran Anil

**Affiliations:** 1 Department of Oral Pathology and Microbiology, Institute of Dental Sciences, Siksha O Anusandhan University, Bhubaneswar, Odisha, India; 2 General Dental Practitioner, Odisha, 757001, India; 3 Centre of Medical and Bio-allied Health Sciences Research, Ajman University, Ajman, Ajman, United Arab Emirates; 4 Basic Medical and Dental Sciences Dept, Ajman University, Ajman, Ajman, United Arab Emirates; 5 Department of Periodontics and Implantology, Institute of Dental Sciences, Siksha O Anusandhan University, Bhubaneswar, Odisha, India; 6 Department of Dentistry, Oral Health Institute, Hamad Medical Corporation, Doha, Doha, Qatar; 7 Qatar University, Doha, Doha, Qatar

**Keywords:** Odontogenic tumour; Ameloblastoma; Ameloblastic carcinoma; immunohistochemistry; Biomarkers

## Abstract

**Background:**

Differentiating between ameloblastoma (AB) and ameloblastic carcinoma (AC) is difficult, especially when AB has atypical cytological characteristics or an uncommon clinical history. This systematic review and meta-analysis aimed to elucidate the differential expression of immunohistochemical markers between AB and AC.

**Methods:**

We conducted a thorough search of PUBMED and SCOPUS according to the Preferred Reporting Items for Systematic Reviews and Meta-Analyses (PRISMA) guidelines to identify cross-sectional studies that compared the expression of immunohistochemical markers in AB and AC. We used a random-effects model to analyze the risk ratios and their corresponding 95% confidence intervals (CIs). The quality of the included studies was assessed using the Newcastle-Ottawa scale. The Egger’s test was used to assess publication bias.

**Results:**

In total, 301 articles were identified. After excluding irrelevant titles and abstracts, 86 articles were selected for full-text review. We categorized the 41 markers into proliferative and non-proliferative markers. Among non-proliferative markers, nuclear markers were differentially expressed in AB and AC. SOX2 was the only marker that significantly differentiated AB and AC, with an RR of -0.19 (CI 0.10-0.36, I2=0).

**Conclusion:**

The current evidence suggests the significance of SOX2 in differentiating between AB and AC, warranting prospective confirmation in well-defined extensive studies. We highlight the paucity of high-quality replicated studies of other markers in this field. Collaborative efforts with standardized techniques are necessary to generate clinically useful immunohistochemical markers.

## Introduction

Odontogenic tumors are a diverse group of lesions that range from hamartomatous or non-neoplastic tissue proliferation to malignancies with metastatic potential. ^1^ These tumors account for less than 2-3% of oral lesions, making them rare and difficult to diagnose without proper experience. ^2^ Although clinical, radiographic, and microscopic features are crucial for diagnosing odontogenic tumors, confirmation may require immunohistochemical findings. ^3^

Ameloblastoma (AB) is a benign epithelial odontogenic tumor that originates in the enamel organ. On the other hand, Ameloblastic Carcinoma (AC) is a malignant epithelial odontogenic tumor that shows histological features of benign ameloblastoma with cytological atypia and rare metastatic potential. ^4^ AC can arise de novo or from its benign counterpart, ^5^ and recent studies have demonstrated the influence of genetic and environmental factors on its pathogenesis. ^6^ Although the clinical and radiological features of AB and AC are similar, cytological atypia is the key differentiating factor between the two tumors. However, in cases where cytologic atypia is more frequent in AB and less frequent in AC, distinguishing them by Hematoxylin and Eosin staining can be difficult. In such cases, immunohistochemical (IHC) intervention is necessary. However, no definitive marker can differentiate AB from AC. Therefore, this study aimed to identify differences in the expression of immunohistochemical markers in AB and AC to understand the biological behavior of the tumors and the potential of these markers to differentiate between AB and AC.

## Methods

This systematic review is based on the Preferred Reporting for Systematic Review and Meta-Analysis (PRISMA). ^7^ The protocol was registered in the **PROSPERO** database (ID: CRD42021285592).

### Search strategy

The PECO format was used to construct the search strategy, where specimens from patients diagnosed with ameloblastic carcinoma (P) were subjected to immunohistochemistry (E) and compared with those diagnosed with ameloblastoma (C). Outcomes (O) assessed included immuno-expression of various markers reported as intensity, proportion of positive cases, and total immunoreactivity scores (IRS). The search used MeSH and keywords from two databases, PUBMED and SCOPUS. Boolean operators like “‘AND’ and ‘OR’” were appropriately used. The reference lists of the selected articles and grey literature were further searched. The time period of The search lasted till 31 ^st^ May 31, 2023.

### Elligibility criteria

Only English literatures fulfilling PECO criteria were included in this study. Missing clinical data,sample sizes in either group less than 3 and ratio of AB:AC greater than 5:1 ^8^ were excluded. Studies lacking clarities among AB,AC and unicystic AB were discarded. Studies which had not specified outcome measures were also excluded.

### Data extraction

Two investigators (SM and SP) independently screened the identified articles initially by title and abstract, followed by full text, considering the inclusion and exclusion criteria. Data were collected in an Excel sheet and included the first author, year of study, study population, sample size, age, sex, IHC markers, proportion of positive cases, intensity of immuno-expression, proliferative index of proliferative markers, and immunoreactivity score (IRS).

### Data analysis

We considered moderate to strong immune expression to be positive. The data were pooled using a meta-analysis. Meta-analysis was conducted using Revman (version 5.4.1). Forest plots were constructed for each reported marker with a Risk Ratio as the outcome measure for the number of positive cases, whereas the mean difference was the outcome measure when comparing IHC scores between AB and AC. Publication bias was assessed using funnel plots in RevMan (version 5.4.1) and the Egger’s test. A sensitivity analysis was also conducted to determine the influence of each study on the results.

### Quality assessment

The New Castle–Ottawa scale ^9^ was used to evaluate the quality of the included studies.

## Results

### Study selection

**Figure 1.**
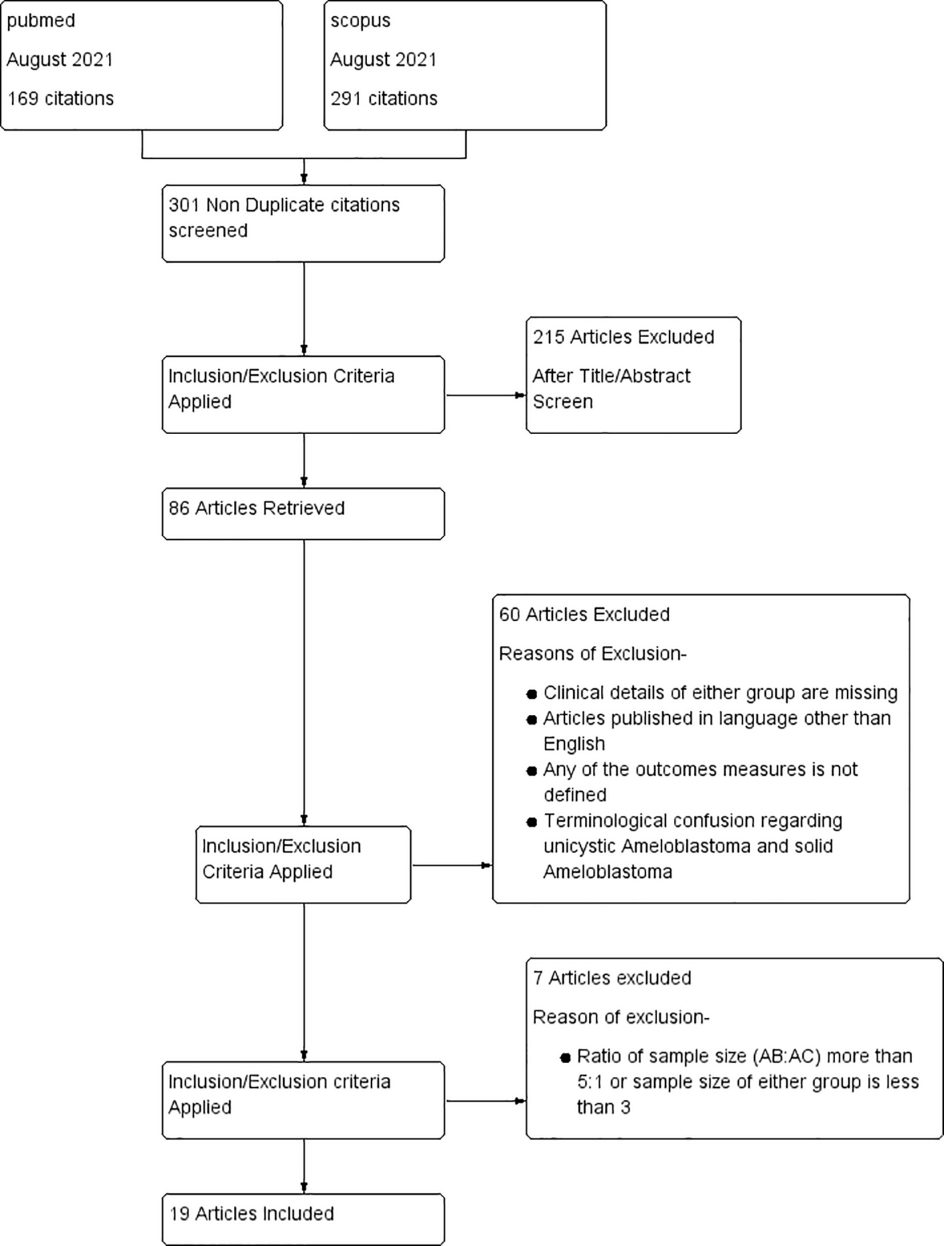
Prisma flowchart.

A total of 463 articles were selected from the two databases in the first phase. After removing the duplicate studies, there were 303 articles. After a further comprehensive evaluation of titles and abstracts, 215 articles were excluded. After a full-text screening, 63 articles were excluded. Six articles were excluded during the data extraction. ^10^^–^^15^ Nineteen articles were included in the systematic review and meta-analysis. A PRISMA flowchart is shown in Figure 1 *.*

### Study characteristics

***Clinical features***

There were 11 studies ^10^^–^^20^ from Asia, three from North America, ^21^^–^^23^ four from South America, ^24^^–^^27^ and one ^28^ from the Australian population. The age ranges in AB and AC were found to be 11–78 years ^11^^,^^13^^,^^18^^,^^20^^,^^22^^,^^25^^,^^28^ and 16–72 years, respectively. ^11^^,^^13^^,^^18^^,^^20^^,^^22^^,^^25^^,^^28^ Male prevalence of 1.1:1 and 1.9:1 were observed in AB and AC, respectively. Mandibles were the predominant sites in both the AB and AC, with mandibular to maxillary ratios of 8.2:1 and 4.4:1, respectively. The included studies used different methods of interpreting immunohistochemistry have been utilized, such as the combinative semiquantitative scoring system, Allred scoring system, immunoreactivity score, automation approach, Klein scoring system, qualitative scoring system, and evaluation of the number of IHC-positive cases. ^29^

***Immunohistochemical features***

Nineteen articles studied 41 markers ^10^^–^^28^ which could be categorized as proliferative and nonproliferative markers. Ki67, ^11^^,^^13^^,^^15^^,^^22^^,^^23^^,^^24^ AgNOR, ^15^^,^^25^ PCNA, ^12^ and p53 ^22^^,^^24^ are all proliferative markers. The non-proliferative markers were further divided into epithelial and stromal markers. Epithelial markers are further subdivided into cell membrane, cytoplasmic, and nuclear markers. The details of these studies are provided in Table 1 (Extended data)

***Proliferative markers***

Four proliferative markers, Ki67, AgNOR, PCNA and P53, were compared between AB and AC by six, ^11^^,^^13^^,^^15^^,^^22^^,^^23^^,^^24^ two, ^15^^,^^25^ one, ^12^ and two authors, ^22^^,^^24^ respectively. The intensities of Ki67 and P53 expression were higher in AC than in AB. ^19^^,^^30^ All studies, except one, ^30^ demonstrated significant overexpression of all four proliferative markers in AC compared to AB.

***Non-proliferative markers***

*Epithelial markers*

Five studies explored five cell membrane markers, ten studies examined 12 nuclear markers, 13 studies explored 28 cytoplasmic markers, and five studies explored seven stromal markers. Epithelial markers are further categorized into cell membrane, nuclear, and cytoplasmic markers, depending on the localization of the antibody.

*Cell membrane markers*

Of the five studies ^17^^,^^21^^,^^22^^,^^23^^,^^28^ on cell membrane markers, the intensity of CD138 was reported to be stronger in AB in only one. The intensities of CD44 ^28^ and nestin ^17^ were stronger in AC. However, the area of staining has not been adequately investigated. The number of CD44-positive cases was higher in the AB group than in the AC group. Similarly, the intensity of CD138 was higher in AB; in contrast, the number of CD138 positive cases was higher in AC. The intensity and number of nestin-positive tissues were higher in the AB group. Even the mean CD138 score, as found in the automation method, was significantly higher in AB than in AC. ^23^ Mean score of CD44 score, as found by Ge et al. was 1.20±0.89 AB and 0.8±0.41 AC. ^17^ Comparative data for CD56 and E-cadherin are not available.

*Nuclear markers*

While there was no difference in SOX2 intensity between AB and AC in Yu ^21^ and Sanjai ^18^ reports, Wafa et al. ^17^ demonstrated a significantly higher intensity in AC than in AB. All three authors ^17^^,^^18^^,^^21^ reported a significant increase in SOX2 immunopositive cases in AC compared to AB. Calretinin was found to be intense in AB as compared to no reactivity in AC, as reported by Amrutha et al. ^19^ Proportion of Calretinin immune-positive cases is contradicted in two studies. ^6^^,^^19^ Intensity of P16, HIF alpha, NF-kb, and ZEB1was found to be higher in AC than in AB. There have been no reports on differences in the number of positive cases. Both the intensity and proportion of immunopositive patients for Twist, OCT4, and Nestin were higher in AC than in AB. There was no difference in the number of immunopositive cases for Maspin and B-catenin. The β-catenin ^19^ immunohistochemical score was not significantly different (0.21) between AB and AC. Allred scoring system (ARS) for ZEB1 was 1.8 and 4 for AB and AC, respectively, with a significant difference (p <0.05). ^20^ ARS for HIF1α ^20^ was 1.5 & 4.6 for AB & AC respectively with a significant difference of <0.01. Loyola et al. ^24^ found the mean quick score for p16 as 7.9±2.6 & 10.3±3.8 in AB and AC, respectively though the difference was not significant. Sanjai et al. ^18^ used an immunoreactive scoring system for SOX2 and found a score of 3.46±4.03 AB and 6.5±3.99 AC. Wafa Khan et al. ^17^ found SOX2 scores of 0.3±0.73 for AB and 5.2±2.14 for AC, respectively. The OCT4 score for the AC group was 5±2.43 AC. Safadi et al. ^13^ used an automation score system for Maspin and found 110.70% and 53.20% for AB and AC, respectively.

*Cytoplasmic markers*

The intensities of Nfkb, ^14^ Bclxl, ^14^ p16, ^24^ and COX2 ^14^ were stronger in AC than in AB. The number of positive cases for ck14, ^8^^,^^19^ ck6, ^13^ck5, ^22^ck17, ^13^CD138, ^13^ and ck19 ^11^^,^^13^ were higher in AB than in AC. The immuno-expression of calretinin in the two studies ^6^^,^^19^ is contradictory. Although the perilipin intensity was lower in AC than in AB, the proportion of positive cases was higher in AC than in AB. Similarly, the adipophilin intensity was higher in the AC group, while the proportion of adipophilin-positive cases was higher in the AB group. There have been no reports on the intensity changes of Snail; however, the proportion of positive patients is higher in AC than in AB. The intensity and proportion of positive cases of CK 18 and MMP2 are more in AC than in AB. The expression of nestin ^28^ and MMP2 was augmented in AC compared to AB during qualitative and quantitative evaluation. CK 7 was not expressed in either the AB or AC. However, the mean immunohistochemical scores for FASN, COX2, Perilipin 1, and adipophilin were higher in AB than in AC. ^26^ Mean scores of P53, P16, CK18 and CK19 were significantly higher in the AC group than in the AB group. ^24^

*Stromal markers*

**Figure 2.**
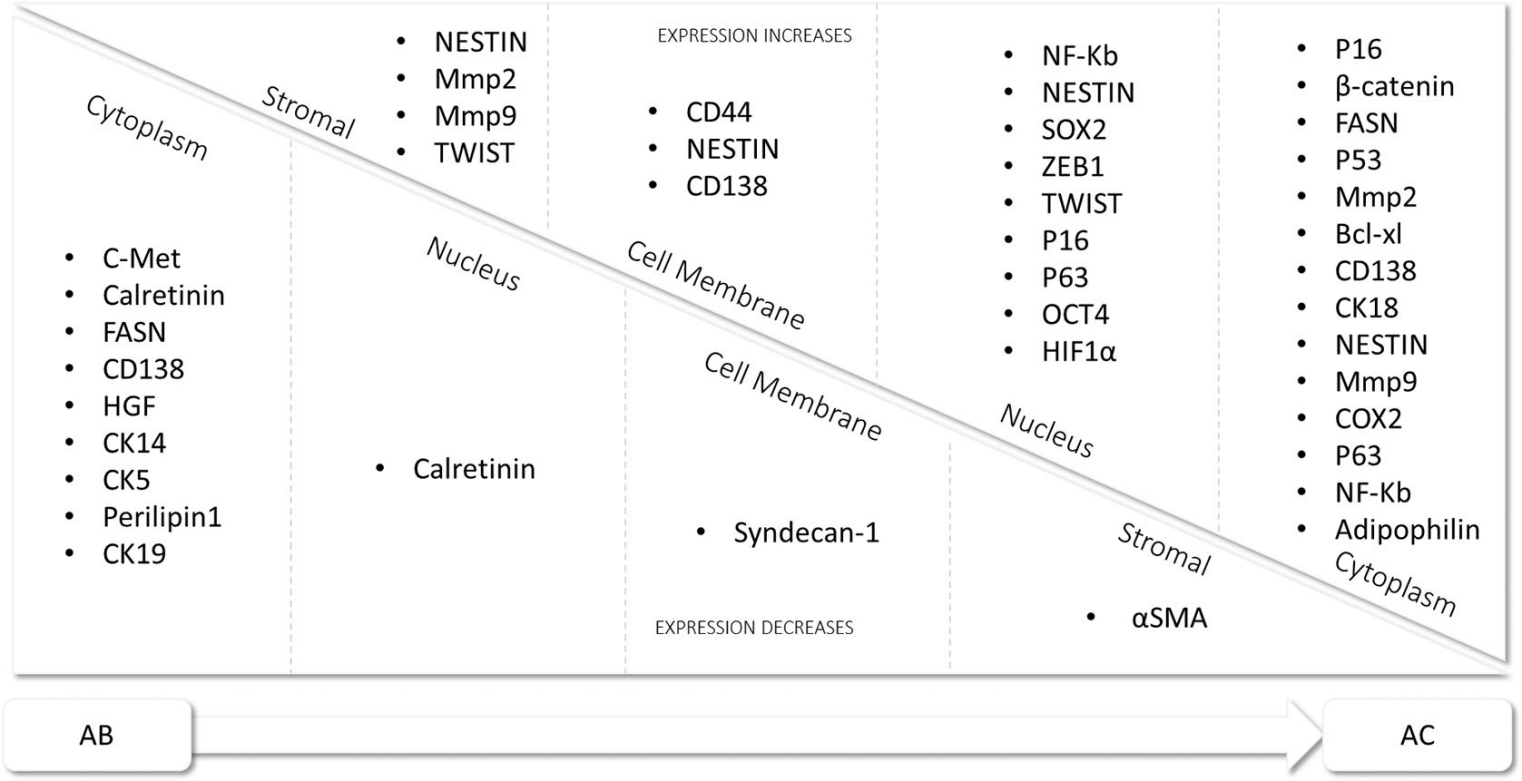
Differential expression of immunohistochemical markers in AB and AC.

The intensity of expression and proportion of immuno-positive cases of MMP9 ^11^ and nestin ^28^ were higher in AC than in AB. The proportion of immunopositive cases of MMP2, ^11^ Twist, ^16^ and CD138 ^28^ was found to be higher in AC than in AB. However, Da Silva et al. ^25^ did not observe any differences in the expression of MMP2 and MMP9. The number of Snail positive ^16^ cases was marginally higher in AB than in AC, and differential expression of immunohistochemical markers in AB and AC are shown in Figure 2.

### Metaanalysis

**Figure 3.**
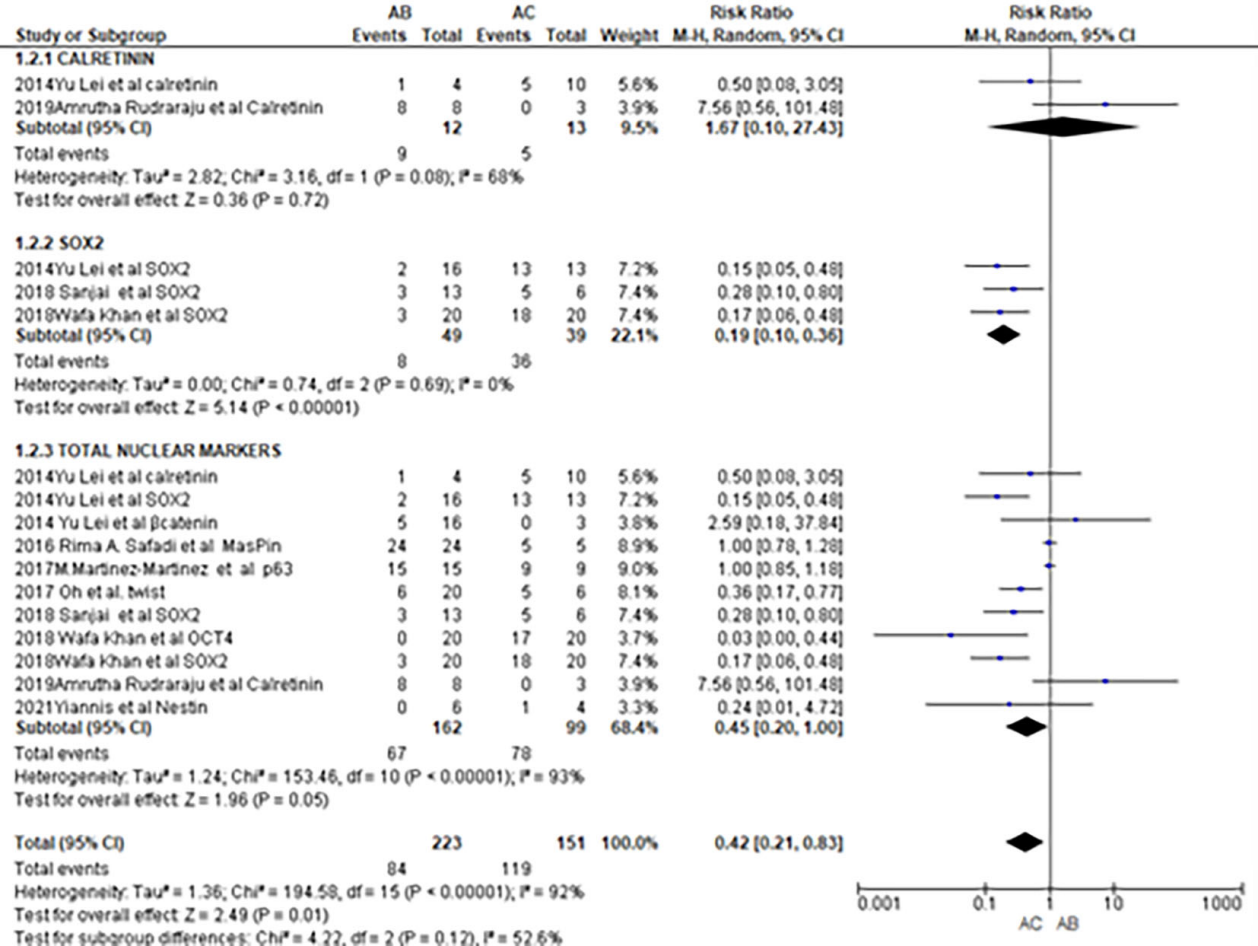
Meta-analysis of nuclear markers.

Meta-analysis of the comparison of IHC scores of four proliferative markers, Ki67, AgNOR, PCNA and P53 between AB and AC did not find any conclusive difference in expression (mean differences = -0.69, 95%CI: -3.34-1.96, p = 0.61). Meta-analysis of the IHC scores of proliferative and nuclear markers was not performed because of the heterogeneity in the reported data. A meta-analysis on the number of cases positive for nuclear markers ^17^^,^^18^^,^^21^ demonstrated that SOX2 has 81% potential in differentiating between AB and AC (RR-0.19; 95%CI -0.10-0.36,p-value of <0. 00001). Altogether, nuclear markers have 55% potential in differentiating between AB and AC (RR-0.45; 95%CI-0.20-1.00;p-value of 0.05). Forest plots depicting the meta-analysis of nuclear markers are shown in Figure 3. Meta-analysis of cytoplasmic markers (RR-0.85, 95% 184 CI: 0.17-4.20, p =0.84) and stromal markers (RR: 0.90, 95% CI: 0.73-1.10, p = 0.29, and Tau2 = 0.02). did not reveal any differential expression between AB and AC.

### Quality assessment and sensitivity analysis

The quality of the studies on the Newcastle–Ottawa scale varied from to 7-8. The sensitivity analysis showed that none of the studies affected the risk ratio of SOX2. Two studies by Yu Lei et al. for β Catenin ^21^ and Rudraraju et al. ^19^ for Calretinin influenced the results to achieve an optimum risk ratio.

### Publication bias

Egger’s test shows significant publication bias exists among the selected articles.

## Discussion

Diagnostic difficulty in ameloblastic neoplasms frequently occurs in one of two ways: either ^23^ the degree of cytologic atypia and loss of ameloblastic differentiation is intermediate, making it difficult to classify the lesion as either Atypical Ameloblastoma (AA) or AC, or ^28^ depending on the degree of cytologic atypia and high-grade transformation, overlapping histological features between AA and AC can be perplexing. Moreover, there are a handful of studies on the proteins involved in the malignant transformation of AB to AC. ^31^ This review will help us understand the differential expression of immunohistochemical markers in AB and AC, as well as to evaluate the diagnostic potential of these markers in differentiating AC from AB.

This systematic review included 19 comparative cross-sectional studies and evaluated the differences in clinical features and immunoexpression of nuclear, cytoplasmic, cell membrane, stromal, and proliferative markers between AB and AC. Minimal differences were identified in the clinical characteristics of patients with AB and AC. The male-to-female ratios in AB and AC were found to be approximately 1:1 and 2:1, respectively, which is in agreement with the global profile of AB ^32^ and AC. ^33^ Moreover mandible was the predominant jaw in both AB and AC. ^34^ Mean age range for both AB and AC patients was from the first decade to the seventh decade.

A previous systematic review evaluated the prognostic implication of immuno-expression of MMP2 and MMP9 in AB and AC, ^35^ and another correlated Ki 67 and p53 expression in AB and AC with clinicopathological features. ^33^ Both these systematic reviews ^33^^,^^35^ reviewed a limited number of markers and did not analyze the comparative immuno-expression of these markers in AB and AC. This study presents the first comprehensive evaluation of the comparative expression of IHC in AB and AC.

Among the 12 nuclear marker studies, a progressive increase in staining intensity and proportion of positive cases was observed with SOX2, Twist, OCT4, and Nestin from AB to AC ( Figure 3). OCT4 and SOX2 are two crucial cancer stem cell markers involved in oncogenic processes and are known to contribute to the aggressive behavior of odontogenic tumors. ^36^^,^^37^ Our results suggest that SOX2 may serve as a prognostic marker for malignant transformation of ameloblastoma. It is plausible that SOX2 is involved in the carcinogenesis of AC, as SOX2 has been shown to coordinate with inflammatory signalling to convert epithelial progenitor cells into invasive squamous carcinoma cells. ^38^ SOX2 has also been shown to significantly distinguish odontogenic tumors from cysts. ^21^

A higher intensity of P63 and Maspin in AC than in AB was observed in this study. The evidence on P63 immuno-expression association with aggressive features of odontogenic cysts in literature is contradictory to this study, ^39^ and generally, loss of p63 has been linked to aggressive behavior in cancer. ^40^ An increase in the intensity of p16 in AC compared to AB has been supported by Khojasteh et al., who reported CpG methylation of p16 in all 18 samples of AC compared to only one case of AB. ^41^ This may suggest that p16 expression is a predisposing factor for the malignant transformation of AB. There was a conflicting result for another nuclear marker, calretinin, whose intensity was reduced in AC compared to AB in one study, ^42^ whereas the reverse was observed in another study. ^43^ Calretinin was significantly associated with AB compared to other odontogenic tumors.

The expression of two membranous markers, CD44 and CD 138, and the cytoplasmic marker perilipin was augmented in AB and reduced in AC. CD44, a family of cell surface glycoproteins, participates in cell-to-cell and cell-to-extracellular matrix adhesions and interactions. ^44^ The expression profile of CD44 is tissue-specific, which is attributed to the tissue-specific distribution of various isoforms of CD44 and is currently inconclusive regarding AB and AC. Reduced expression of CD44 has also been observed in oral cancer. ^36^^,^^37^^,^^45^ Loss of function of CD44 in AC must be confirmed through further research focused on variants of CD44 and its interaction with several related molecules. CD 138 (syndecan 1) is another membrane protein that is functionally similar to CD44. CD138 expression was reduced in head and neck cancer, gastric cancer, and colorectal cancer compared with the adjacent normal epithelium. ^46^ Therefore, the present finding of reduced expression in AC compared to AB is supported by the existing literature. However, a limited number of studies have not provided conclusive evidence. Perilipins are a group of proteins related to the surface of lipid droplets. Perilipin expression is upregulated in renal cell carcinoma, gastric cancer, and non-small cell lung cancer, and its increased expression is associated with improved survival. However, breast cancer, oral cancer, hepatocellular cancer, and colorectal and pancreatic cancers have decreased expression of perilipin, which is associated with poor survival and increased invasion. ^35^^,^^47^^–^^49^ Increased expression of perilipin 1 in AC was observed compared to that in AB. ^27^ Other cytoplasmic markers, Nestin, FASN, NF-kB, BCL-XL, p63 Adipophilin, and COX2, were shown to be overexpressed in AC as compared to AB. The present finding of higher COX2 expression in AC than in AB, supported by the previous evidence of higher expression of COX2 in OKC compared to AB ^50^^–^^52^ association with high recurrence and low disease-free survival in AB, ^53^ may suggest the involvement of this molecule in the biological aggressiveness of jaw tumors. COX 2 probably maintains tumor growth and facilitates invasiveness by interfering with apoptosis, cellular proliferation, and angiogenesis, and hence could be a potential biomarker. However, further studies are required to clearly delineate this relationship. Nestin is an intermediate filament of the cytoskeleton, and its expression is related to tooth development and dentin repair. The negative expression of Nestin in AB is also supported by Fujita et al. ^54^ NF-kB is a molecule of Protein Kinase B (AKT pathway) and is shown to be a putative regulator of local invasiveness of AB. ^55^ Together with the present finding of increased expression of NF-kB in AC compared to AB suggests further exploration of the predictive potential of this marker in malignant transformation of AB. Although p63 was shown to differentiate between AB and AC, as reported in this study, previous evidence suggested no discriminatory potential of this marker between odontogenic cysts and odontogenic tumors. ^56^ The diversity in the expression of P63 in odontogenic tumors has been reported by Alsaegh et al. ^57^ P63 is also registered for both cytoplasmic and nuclear localization. ^22^ Although BCL-XL has never been studied in any malignant odontogenic tumors, other anti-apoptotic molecules such as Bcl-2 have been studied in AB, which is known to be highly expressed in AB and associated with recurrence. ^58^ Matrix, which are involved in extracellular matrix degradation, play a vital role in the local invasion of ameloblastomas. The present findings implicate a possible role of MMP2 in the malignant transformation of AB to AC, as observed in the stringer intensity in AC compared to AB. This finding is supported by another systematic review conducted to show the difference in MMP expression between AB and AC. However, there was no difference in MMP9 expression in either tumor, which is also supported by Zhou et al. ^35^ Calretinin and perilipin were expressed less in AC than AB. Thus, we suggest the presence of these two molecules. Evidence of the differentiating potential of calretinin between a dentigerous cyst, OKC, and ameloblastoma The present findings focus on the differentiating potential of Calretinin in AB and AC. ^59^^–^^61^ HGF, c-Met, CK7, CK7, CK7, CK14, CK14, CK14, CK19, MMP9, CK5, CK8, β-catenin, Snail, and EGFR did not show any difference in staining intensity between AB and AC.

Proliferative markers, such as Ki 67, ^11^^,^^13^^,^^15^^,^^22^^–^^24^ P53, ^22^^,^^24^ PCNA, ^12^ and AgNOR, ^22^^,^^25^ were shown to differentiate between AB and AC in individual studies. However, the pooled meta-analysis findings were unreliable, which may have occurred because of the strong heterogeneity in sample size and score-determining techniques. The proliferative index of Ki67 was shown to be higher in secondary AC than in primary AC. ^62^ In ameloblastoma, Ki67 is observed in peripheral ameloblast-like cells, whereas in AC, it is distributed in central stellate reticulum-like cells and peripheral ameloblast-like cells. The peripheral location of Ki 67 in AB was also reported by Sathi et al. ^62^ Ki67 was also reported to be highly intense in clear cell odontogenic carcinoma. ^24^

This study had certain limitations. First, all the included studies were retrospective. The use of different antibodies in immunohistochemistry may be a potential confounding factor for all IHC studies. The sample sizes of the included studies for AC were significantly smaller, although we selected studies that chose an optimum ratio for case-control studies of 5:1. ^8^ High statistical heterogeneities were found among these studies, which limited the scope of this systematic review and meta-analysis. Selective reporting, such as the absence of staining intensity, lack of proportion of positive cases, and demographic details in many studies, obscure the comparison between AB and AC.

## Conclusions

This systematic review and meta-analysis identified differential expression of SOX2 in AB and AC, which may be considered the most promising marker to not only differentiate AB from AC, but also a plausible risk factor for the malignant transformation of AB. Furthermore, our study identified potential immunohistochemical biomarkers that may be worthy of validation in well-designed, large, prospective trials. A panel of molecules engaged in several pathways may be able to discriminate with higher sensitivity and specificity than individual markers, because of the complexity of the transformation process. Based on our results, marker panels with potential discriminative values were created.

## Ethical approval and consent

Ethical approval and consent were not required.

## Data Availability

All data underlying the results are available as part of the article and no additional source data are required. Figshare: Differential expression of immunohistochemical markers in ameloblastoma & ameloblastic carcinoma: a systematic review and meta-analysis of observational studies, DOI: https://doi.org/10.6084/m9.figshare.25836703.v3. ^63^ This project contains the following extended data:
• PRISMA Flowchart for The Systematic Review •
Table 1. Characteristics of Study • Risk of Bias Assessment • Completed PRISMA checklist Data are available under the terms of the Creative Commons Zero “No rights reserved” license (CC0). Figshare: Checklists for Differential expression of immunohistochemical markers in ameloblastoma & ameloblastic carcinoma: a systematic review and meta-analysis of observational studies, DOI: https://doi.org/10.6084/m9.figshare.25836703.v3. ^63^
