# Supplementary material for: Differential Expression of Immunohistochemical Markers in Ameloblastoma & Ameloblastic Carcinoma: A Systematic Review and Meta-analysis of observational studies
Source: F1000Res. 2024 May 31;13:557. [Version 1] doi: 10.12688/f1000research.149861.1 (PMC11287113; doi:10.12688/f1000research.149861.1)
Supplement: Peer review history

# Reviewer report: Reviewer response for version 1

**DOI:** 10.5256/f1000research.164369.r301590

**Published:** 2024-07-29

**Authors:** Maya Ramesh

**Affiliations:** 1 Department of Oral Pathology, Department of Pedodontics, Vinayaka Mission’s Sankarachariyar Dental College, Salem, Tamil Nadu, India

The article is a comprehensive one on Immunohistochemical markers that can be used to differentiate between ameloblastoma and ameloblastic carcinoma.

 The objectives of the study are clearly stated, and methodology is clear for anyone else to replicate it . 

 Statistical analysis and interpretations are very clear. Figure 2 shows the markers' staining intensity and the area where it is seen very clearly.

 This study provides a panel of markers to differentiate between Ameloblastoma and Ameloblastic carcinoma. 

 But in this Systematic review and Meta analysis , number of cases of ameloblastic carcinomas were comparatively less and the IHC markers used in different studies were not made by the same manufacturer. These were the limitations as identified by the author also.

Are the rationale for, and objectives of, the Systematic Review clearly stated?

Yes

Is the statistical analysis and its interpretation appropriate?

Yes

If this is a Living Systematic Review, is the ‘living’ method appropriate and is the search schedule clearly defined and justified? (‘Living Systematic Review’ or a variation of this term should be included in the title.)

No

Are sufficient details of the methods and analysis provided to allow replication by others?

Yes

Are the conclusions drawn adequately supported by the results presented in the review?

Yes

Reviewer Expertise:

Ameloblastoma, Dental fluorosis, Oral cancer

I confirm that I have read this submission and believe that I have an appropriate level of expertise to confirm that it is of an acceptable scientific standard.

# Reviewer report: Reviewer response for version 1

**DOI:** 10.5256/f1000research.164369.r288916

**Published:** 2024-06-24

**Authors:** Lilies Dwi Sulistyani, Lilis Iskandar

**Affiliations:** 1 Department of Oral and Maxillofacial Surgery, Universitas Indonesia, Depok, West Java, Indonesia; 2 Oral and Maxillofacial Surgery, Universitas Indonesia (Ringgold ID: 64733), Depok, West Java, Indonesia

I think this is a really great article. The rationale and objectives of the systematic review are clearly stated, and the methods are clearly elaborated. However, it would have been greater if the IHC expressions, both the proliferative and non-proliferative, were pooled and summarized in a well-organized table to showcase the results to help readers to analyze and draw conclusions from the data.

 In the article, it was reported that Egger's test shows significant publication bias exists among the selected articles, yet there were no  further discussion on that issue. 

 In the discussion and conclusion sections, SOX2 were highlighted as a promising differentiating marker between AB and AC, also a predicting marker of malignant transformation of AB. This statement is a bit confusing in clinical setting as the cutting point was not clearly explained when the positive SOX2 IHC expression should be considered a diagnostic marker for AC, and when to consider it as a predictive marker of malignant transformation of AB.

 Yet this study truly intrigue the need of a controlled prospective study to develop a standard IHC panels to clearly define AB and AC.

Are the rationale for, and objectives of, the Systematic Review clearly stated?

Yes

Is the statistical analysis and its interpretation appropriate?

I cannot comment. A qualified statistician is required.

If this is a Living Systematic Review, is the ‘living’ method appropriate and is the search schedule clearly defined and justified? (‘Living Systematic Review’ or a variation of this term should be included in the title.)

Not applicable

Are sufficient details of the methods and analysis provided to allow replication by others?

Yes

Are the conclusions drawn adequately supported by the results presented in the review?

Partly

Reviewer Expertise:

oral and maxillofacial surgery

We confirm that we have read this submission and believe that we have an appropriate level of expertise to confirm that it is of an acceptable scientific standard.
